# Selective Protection of Human Liver Tissue in TNF-Targeting of Cancers of the Liver by Transient Depletion of Adenosine Triphosphate

**DOI:** 10.1371/journal.pone.0052496

**Published:** 2012-12-18

**Authors:** Timo Weiland, Kathrin Klein, Martina Zimmermann, Tobias Speicher, Sascha Venturelli, Alexander Berger, Heike Bantel, Alfred Königsrainer, Martin Schenk, Thomas S. Weiss, Albrecht Wendel, Matthias Schwab, Michael Bitzer, Ulrich M. Lauer

**Affiliations:** 1 Department of Internal Medicine I, Medical University Hospital, Tuebingen, Germany; 2 Dr. Margarete Fischer-Bosch Institute of Clinical Pharmacology, Stuttgart, Germany; 3 Department of Biology, Institute of Cell Biology, ETH Zürich, Switzerland; 4 Department of Gastroenterology, Hepatology and Endocrinology, Hannover Medical School, Hannover, Germany; 5 Department of General, Visceral & Transplant Surgery, University Hospital, Tuebingen, Germany; 6 Center for Liver Cell Research, University Hospital, Regensburg, Germany; 7 Interfaculty Center for Pharmacogenomics and Drug Research (ICEPHA), University of Tuebingen, Germany; 8 Department of Clinical Pharmacology, Institute of Experimental and Clinical Pharmacology and Toxicology, Medical University Hospital, Tuebingen, Germany; Medical University Graz, Austria

## Abstract

**Background:**

Tumor necrosis factor alpha (TNF) is able to kill cancer cells via receptor-mediated cell death requiring adenosine triphosphate (ATP). Clinical usage of TNF so far is largely limited by its profound hepatotoxicity. Recently, it was found in the murine system that specific protection of hepatocytes against TNF's detrimental effects can be achieved by fructose-mediated ATP depletion therein. Before employing this quite attractive selection principle in a first clinical trial, we here comprehensively investigated the interdependence between ATP depletion and TNF hepatotoxicity in both *in vitro* and *ex vivo* experiments based on usage of primary patient tissue materials.

**Methods:**

Primary human hepatocytes, and both non-tumorous and tumorous patient-derived primary liver tissue slices were used to elucidate fructose-induced ATP depletion and TNF-induced cytotoxicity.

**Results:**

PHH as well as tissue slices prepared from non-malignant human liver specimen undergoing a fructose-mediated ATP depletion were both demonstrated to be protected against TNF-induced cell death. In contrast, due to tumor-specific overexpression of hexokinase II, which imposes a profound bypass on hepatocytic-specific fructose catabolism, this was not the case for human tumorous liver tissues.

**Conclusion:**

Normal human liver tissues can be protected transiently against TNF-induced cell death by systemic pretreatment with fructose used in non-toxic/physiologic concentrations. Selective TNF-targeting of primary and secondary tumors of the liver by transient and specific depletion of hepatocytic ATP opens up a new clinical avenue for the TNF-based treatment of liver cancers.

## Introduction

Systemic use of the highly potent antineoplastic cytokine tumor necrosis factor (TNF) is highly limited *due to the finding that TNF's pleiotropic functions induce serious systemic side effects*, in particular high grade liver toxicity [1], [2]. Therefore, systemic administration of TNF was replaced in selected tumor entities successfully by regimens only employing local perfusions of extremities with TNF [3], [4]. However, such strategies remained elusive for isolated hepatic perfusion (IHP) used for the treatment of malignancies of the liver [5]–[8].

**Figure 1:**
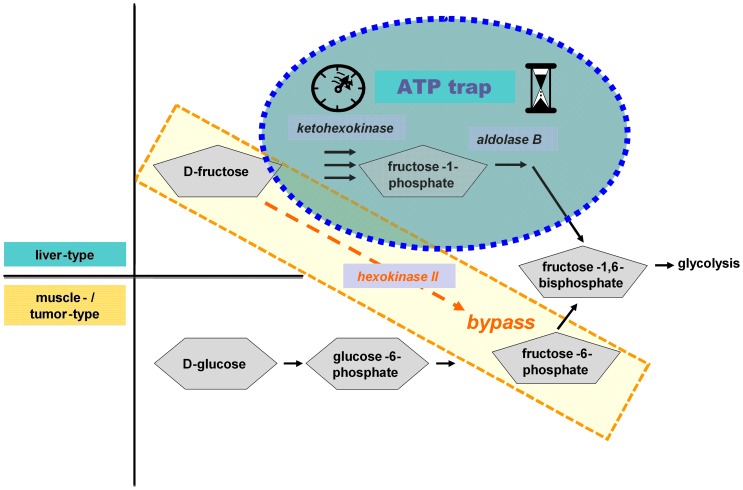
Selective protection from TNF-mediated cell death by fructose-loading: only hepatocytes exhibit a *transient* ATP depletion. Increased expression of hexokinase II (HKII) in tumor cells results in a muscle-type metabolism of fructose (lower part of the picture) which does not encompass any depletion or trapping of ATP. In contrast, hepatocytes (upper part of the picture) utilize the enzymes ketohexokinase (KHK) and aldolase B (aldoB) and thereby a liver-type metabolism of fructose. As a result, there is a very rapid ATP-dependent conversion of fructose via KHK to fructose-1-phosphate acting as a phosphate sink dramatically bringing down cellular levels of ATP.

Recently, TNF-induced hepatocytic apoptosis was recognized as a highly ATP-dependent process in a murine model [9]. Furthermore, there is evidence that fructose leads to ATP depletion exclusively in hepatocytes. As a functional consequence, hepatocytes (in contrast to their malign counterparts) exhibit protection towards TNF-induced apoptosis [9], [10]. This biological difference between hepatocytes and malignant cells was ascribed to a transformation-related overexpression of hexokinase II (HKII) leading to a bypass of the hepatocytic-specific fructose catabolism [10]. In presence of fructose, the critical liver-specific enzymes ketohexokinase (KHK) and aldolase B (AldoB) establish a reversible, hepatocytic-specific ATP trap [11]. Overexpression of HKII bypasses this sink *so that* fructose is converted preferentially into fructose-6-phosphate and metabolized via “muscle-type” glycolysis without affecting cellular ATP levels (depicted in detail in Figure 1).

On the molecular level, *this fructose-mediated hepatocyte-specific protective effect towards TNF* is most likely achieved by ADP degradation products, which accumulate in form of adenosine. Recently, *it was shown that adenosine provokes an autocrine adenosine 3′,5′-cyclic monophosphate response*, negatively affecting TNF-induced activity of c-Jun-N-terminal kinase (JNK) in a protein kinase A-dependent manner [12].

In our study, we investigated the possibility to transfer this approach from murine data to the human system with regard to fructose-mediated transient ATP depletion and thereby effectuating the selective disarmament of TNF's destructive hepatocytic properties. To this end, we utilized (i) cultured primary human hepatocytes (PHH) and, (ii) precision-cut slices of healthy human liver tissues *versus* malignant human liver tissues derived from patients' liver resectates in a translational e*x vivo* approach. In addition to previously conducted murine experimentation, w*e were now able to translate gained knowledge on fructose-mediated hepatic protection into the human physiological context providing the basis for future phase I/II clinical studies*.

## Materials and Methods

### Ethical statement

Cultured, primary human hepatocytes (PHH) are processed from freshly taken liver specimen obtained under liver surgery. Accordingly, PHH do not constitute a cell line; PHH are primary cells. PHH from different donor patients were provided by T.S. Weiss with informed patient consent with respect to taking the samples according to the guidelines of and approved by the charitable state-controlled Human Tissue & Cell Research Foundation, HTCR (for direct information please go to: http://www.htcr.de/english/supply.html), and by A. Königsrainer and M. Schenk (Dpt. of General, Visceral & Transplant Surgery, University Hospital, Tuebingen, Germany) with informed patient consent with respect to taking the samples approved by the local Ethics Committee (Ethik-Kommission an der Medizinischen Fakultät der Eberhard-Karls-Universität und am Universitätsklinikum Tübingen/Ethic commission of the medical faculty of the Eberhard-Karls-University and the University Clinic Hospital Tuebingen). Participants did provide their written informed consent to participate in this study. The Tuebingen ethics committee approved this consent procedure and the Tuebingen ethics committee did not raise any objections against this study. Human liver and liver tumor resectates were obtained with informed patient consent from the Dpt. of General, Visceral & Transplant Surgery, University Hospital, Tuebingen, Germany, according to the guidelines of the local Ethics Committee. Participants did provide their written informed consent to participate in this study (i.e., donation of surgical specimen to the Tuebingen tumor and normal tissue bank). This process was documented by signing the respective patient information. The Tuebingen ethics committee approved this consent procedure and the Tuebingen ethics committee did not raise any objections against this study (Ethik-Kommission an der Medizinischen Fakultät der Eberhard-Karls-Universität und am Universitätsklinikum Tübingen/Ethic commission of the medical faculty of the Eberhard-Karls-University and the University Hospital Tuebingen). No research was conducted outside of our country of residence.

### Cell culture

Primary human hepatocytes were cultured in DMEM supplemented with 100 U/ml penicillin/streptomycin (Serva, Heidelberg, Germany), 18.8 µg/ml hydrocortisone (Merck, Darmstadt, Germany) and 1.68 mU/ml insulin (Novo Nordisk, Bagsvaerd, Denmark). Tumor cell lines were cultured in DMEM supplemented with 10% fetal calf serum. All cells were cultured in a humidified incubator with 37°C and 5% CO_2_. HepG2 were obtained from ATCC, Hep3B and PLC/PRF/5 were obtained from ECACC and HuH7 were obtained from Riken Gene Bank. Cell identities were tested by DNA typing (DSMZ, Braunschweig, Germany). All cells were tested to be negative for mycoplasma contamination.

### Precision-cut tissue slicing

Slicing of tissue samples started within one hour of resection on a vibratome VT1200S (Leica, Wetzlar, Germany) in ice cold oxygen-saturated Krebs-Henseleit buffer (KHB) containing 25 mmol/l glucose (Merck, Darmstadt, Germany), 25 mmol/l NaHCO_3_ and 10 mmol/l HEPES (Carl Roth, Karlsruhe, Germany) after storage in Custodiol (Franz Köhler Chemie, Alsbach, Germany) [21]. Slices were incubated in oxygenated William's E medium containing 25 mmol/l glucose and 50 µg/ml gentamycin (Lonza Bioscience, Verviers, Belgium) in an oxygenated atmosphere (80% oxygen, 5% CO_2_).

### Substances

Recombinant human TNF alpha was purchased from Innogenetics (Ghent, Belgium). Fructose and ActinomycinD (ActD) were purchased from Sigma-Aldrich (Taufkirchen, Germany).

### Treatment of cells and human tissue slices

Cells and slices were pretreated with 50 mM fructose for 30 min and with 1 µg/ml ActD 15 min before administration of 100 ng/ml TNF. Caspase assays were performed after 8 hours, LDH release and cytokeratin 18-cleavage assays were performed 24 hours after TNF treatment.

### Cytotoxicity assays

The percentage of lactate dehydrogenase (LDH) release was determined using the LDH Mono-P assay (Analyticon, Lichtenfels, Germany) calculated as the ratio supernatant/(supernantant + lysate). In tissue slices, the amount of LDH being released into supernatant was determined and normalized to protein content of individual slices. Caspase activities were determined by incubation with 50 µM of substrate *N*-acetyl-Asp-Glu-Val-Asp-aminomethylcoumarin (Ac-DEVD-AMC; Biomol, Hamburg, Germany) in assay buffer (50 mM HEPES, pH 7.4, 1% sucrose, 0.1% CHAPS, 10 mM DTT). The substrate cleavage was measured kinetically by spectrofluorimetry. Caspase activity was determined as the slope of the resulting linear regressions and expressed in arbitrary fluorescence units per minute. Cytokeratin 18-cleavage was determined in the supernatant of tissue slices using the M30 CytoDEATH ELISA kit (Peviva, Bromma, Sweden) according to the manufacturer's instructions.

### ATP determination

ATP content was measured using the CellTiter-Glo Luminescent Cell Viability Assay (Promega, Mannheim, Germany).

### Quantitative real-time PCR

High quality total RNA (0.5 µg) was reverse transcribed using Reverse Transcription Kit and random hexamers (Applied Biosystems, Darmstadt, Germany) according to the manufacturer's instructions. mRNA expression was measured using TaqMan 7500 or 7900HT from Applied Biosystems using specific predeveloped assays for aldolase B (Hs01554887_m1), hexokinase II (Hs00606086_m1) and ketohexokinase (Hs00240827_m1) versus standard curves of pCMV6-XL vectors containing cDNAs of the corresponding genes (purchased from Origene, Rockville, USA). 18S RNA (4308329, Applied Biosystems) was used for normalization.

### Statistics

Experiments were done according to the availability of specimens and reproduced at least 5 times. All data are depicted as fold change to control, which is set to 1. Error bars indicate mean ± SEM. Statistical differences were determined by an unpaired t-test. All statistics were calculated using the program GraphPad Prism 4.01 (GraphPad Software Inc.) and a p value <0.05 was considered as being significant.

## Results and Discussion

### Transient depletion of cellular ATP achieved in primary human hepatocytes by using physiological concentrations of fructose

**Figure 2:**
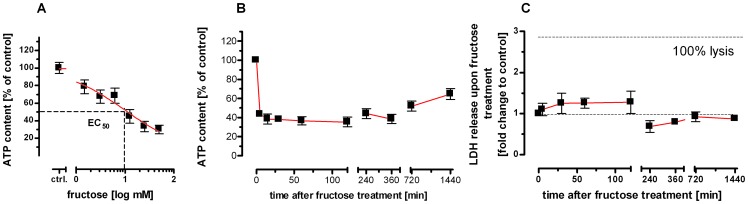
Fructose-loading induces *transient* ATP depletion in primary human hepatocytes. (**A–C**) PHH of human donors were treated with increasing concentrations of fructose (**A**) or treated with a fixed concentration of 50 mM fructose (**B**); ATP content was determined after 30 min (**A**) or at varying time points as indicated (**B**); LDH release of PHH after incubation with 50 mM fructose was determined after indicated time points (**C**). Data are given as mean ± SEM.

When compared to untreated controls, the ATP concentration of PHH *was found to be decreased in a concentration-dependent manner* after 30 min of incubation with fructose, exhibiting a mean reduction to 30% of control at a concentration of 50 mM (Figure 2A, rightmost data point; compilation of six human donors). Kinetically, ATP was found to be depleted within 5 min (Figure 2B) to a mean minimum of ∼40% compared to untreated controls. This ATP depletion was spontaneously reversible as seen by the rise of the ATP content at 120 min and thereafter (Figure 2B, compilation of eight human donors). Thus, cellular energy stores of hepatocytes underwent a constant recovery over the next couple of hours leaving a transient depletion window. Importantly, during fructose-mediated transient depletion of cellular ATP, we did not find any influence on hepatocytic viability as indicated by absence of any significant increase in LDH release before and after initiation of fructose treatment (Figure 2C, compilation of data of four human donors). Of note, any culturing of PHHs beyond our observation time of 1440 min (i.e., 24 hours) has been shown to result in a significant rise of LDH release into cell culture supernatants (our unpublished results) which indicates a profound percentage of disintegrated PHHs beyond the 24 hour threshold. Therefore, “long-term” results concerning PHH viability beyond 24 hours are not feasible in this context. Concurrently, our further basic characterization of PHH cultures also has shown that the ATP contents of these primary cells decrease slowly already within the first 24 hours of testing. Therefore, it is not possible to retain the same ATP levels (100%; measured at the start/initiation of these experiments) throughout 24 hours of PHH culturing. However, the documented recovery of up to 60% of the initial ATP level without any significant release of LDH indicates that the impact of fructose on ATP levels is highly reversible and that hepatocytes are able to cope with this transient decrease in cellular ATP within a limited range of culturing time (i.e., within the 24 hour time span).

Hence, introducing hepatic protection towards TNF in isolated hepatic perfusion (IHP) seems to be feasible from a clinical point of view. In a study by Cortez-Pinto *et* *al*., healthy volunteers as well as patients with non-alcoholic steatohepatitis (NASH) were bolus-injected with fructose. Subsequently, ATP depletion was continuously monitored by NMR spectroscopy for up to 60 min. Both healthy volunteers as well as NASH patients were found to exhibit a rapid (at 12 min after fructose injection) and transient decrease of cellular ATP (restoration of hepatic ATP stores being detected at 60 min post fructose injection) [13]. Therefore, this work by Cortez-Pinto *et* *al*. will be instrumental in monitoring both the time course and the extent of ATP depletion in future phase I/II clinical trials to be performed on patients exhibiting primary and secondary cancers of the liver.

Also other groups observed such a rapid depleting effect on ATP within 30 min when a dose of up to 250 mg fructose/kg body weight was applied in healthy volunteers. Consistent with our experimental results, no detrimental effects on the liver (e.g., with regard to elevated transaminases) were described for such fructose-loading procedures [13]–[17].

### Upregulation of hexokinase II and abrogation of hepatic-type fructose metabolism in liver tumor tissues

It recently was shown in hepatic tumor cell lines that during the process of malignant transformation a profound upregulation of hexokinase II (HKII) occurs which could explain *that the physiological phenomenon of hepatic ATP trapping is bypassed in tumor cells* [10]. To further examine this mechanism we used freshly resected specimens of human liver tissues, i.e. cells which are not subject to any potential artifacts associated with the process of immortalization. Comparing the transcriptional levels of HKII, AldoB, and KHK, the mean level of HKII transcription was found to be increased 7.6-fold in human liver tumors in comparison to non-tumorous primary human liver tissues being normalized to factor 1 (Table 1). In contrast, AldoB and KHK were found to be decreased in the majority of examined tumor tissues and tumor cell lines (mean fold changes <1 in comparison to non-tumorous primary human liver tissues being normalized to factor 1) (Table 1). According to the Warburg hypothesis on acquired alterations in metabolism during progression of malignancies, also cancers of the human liver may accommodate to an environment characterized by lack of nutrients and oxygen via an altered metabolic equipment, in particular in their non-oxidative, i.e. glycolytic energy metabolism [18]. Therefore, overexpression of HKII and thereby altered fructose metabolism in primary or secondary cancers of the liver may serve as a tool for establishing selective hepatic protection in TNF-based tumor therapy by means of an adjunctive fructose administration.

**Table 1:** Upregulation of hexokinase II and muting of hepatic-type fructose metabolism in liver tumor tissues.

mRNA expression level in tumor tissue of ± SEM	HKII	AldoB	KHK
**Mean fold change to liver tissue**	**7.6**±2.32	**0.46**±0.23	**0.47**±0.18
**Mean fold change to hepatic tumor cell lines**	**34.22**±33.67	**0.8**±1.03	**0.93**±0.86

Expression profiles of HKII, AldoB, and KHK in human tumor tissues derived from 10 donor patients were determined by quantitative real-time PCR. Signals were normalized to 18S RNA and were derived for each individual sample as fold induction in comparison to the mean signal of six human non-tumorous hepatic tissue samples, being set to 1. For each enzyme the mean expression ± SEM level is given calculated from averaged donors.

### TNF-cytotoxicity is disabled by fructose-mediated ATP depletion in PHH

**Figure 3:**
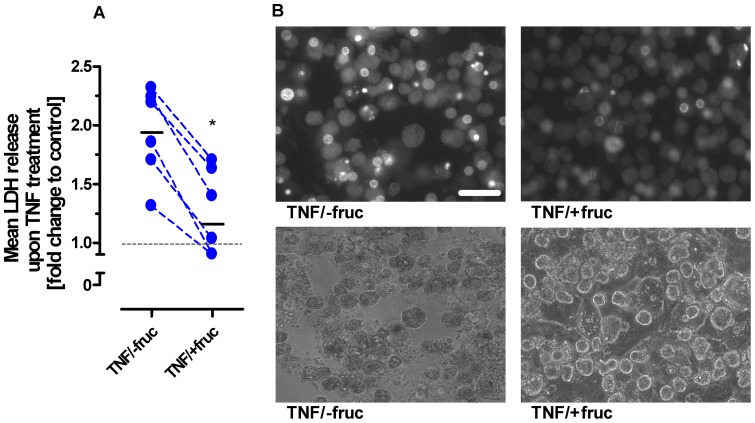
Fructose pretreatment attenuates TNF-induced hepatocytic cell death. Cultured PHH of six different donors were treated with 400 ng/ml ActD alone or in combination with 100 ng/ml TNF and 50 mM fructose as indicated. Cytotoxicity was determined after 24 hours by LDH release assay depicted as mean fold change to untreated control, being set to 1 (*: p<0.05, unpaired t-test). (**B**) Pictures of PHH were taken 12 hours post treatment and illustrate TNF-induced apoptotic condensation of nuclei in dependence of fructose-loading (+/−fruc) (upper panel: Hoechst staining) and membrane blebbing (lower panel: phase contrast microscopy). White bar indicates 10 µM.

Next, consequences of hepatic ATP depletion were assessed on the level of cytotoxicity and caspase activation in human primary hepatocytes. As surrogate marker of membrane integrity, the amounts of LDH release upon exposure to 100 ng/ml TNF alone (100 ng/ml; depicted as −fruc in Figure 2A) or to a combined fructose pretreatment (15 min; +fruc in Figure 2A)/TNF treatment (50 mM fructose; 100 ng/ml TNF) were determined in human donor-derived PHH samples. Throughout these experiments, sensitization to TNF-induced cell death was enhanced by addition of the RNA synthesis inhibitor actinomycin D (ActD; 1 µg/ml), which blocks the up-regulation of any protective survival genes [19]. By examination of PHH derived from resected human liver specimens (Figure 3A), levels of mean TNF-induced LDH release were all found to be attenuated markedly in the course of fructose-loading. In accordance with this functional finding, morphological changes being typical for the TNF-induced apoptotic mode of cell death (e.g., archetypical nuclear condensation and membrane blebbing) were also found to be substantially abrogated by fructose pretreatment (Figure 3B). As exemplified on PHH several apoptotic phenomena like (i) condensation of nuclei (Figure 3B, upper left panel; Hoechst staining) as well as (ii) blebbing of tumor cell membranes (Figure 3B, lower left panel; phase contrast microscopy) clearly were detected in response to TNF treatment alone; however, pretreatment with fructose unanimously led to a nearly complete abrogation of these TNF-induced phenomena (Figure 3B, upper and lower right panels). In prior work, it already has been shown that in hepatocytes TNF-mediated activation of NF-kappa B is abrogated in the course of our regime of concurrent employment of ActD or CPT, imposing a general inhibition on hepatic transcription [20]. Thereby, hepatocytes undergoing a combinatorial treatment with either ActD/TNF or CPT/TNF do not have any possibility to express sufficient amounts of anti-apoptotic proteins such as cFLIP or XIAP whose expression could have been triggered by TNF-induced NF-kappa B-mediated signaling. Accordingly, hepatocytes undergoing fructose-mediated depletion of ATP in presence of either ActD/TNF or CPT/TNF are not able to counteract inhibition of TNF-mediated hepatocytic death via activation of pro-survival effector pathways, such as activation of NF-kappa B signaling [20].

### Abrogation of TNF-induced cell death under ATP depleting conditions in human liver tissues

**Figure 4:**
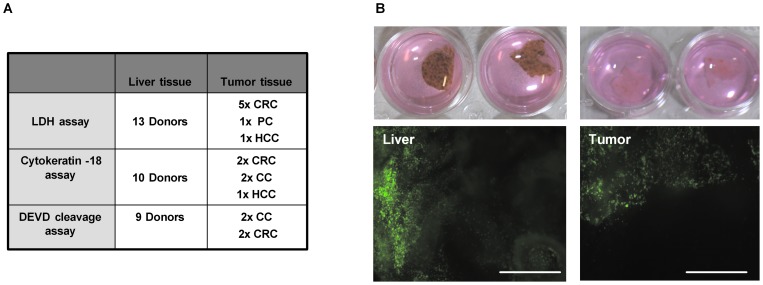
Summary of examined liver tissue and of corresponding tumor tissue specimens and vitality test. Types of tissue samples used for generating data in Figure 4: hepatocellular carcinoma (HCC), colorectal carcinoma (CRC), pancreatic carcinoma (PC), and cholangiocarcinoma (CC) (**A**). As examples, precision-cut slices of human liver tissue (**B; left column**) and human tumor tissue (**B; right column**) were infected with a GFP marker gene encoding adenoviral vector (AdV-GFP, MOI 1) one hour after tissue cutting to determine the vitality of tissue slices cultured in 24-well plates. Pictures were taken 24 hours post infection to determine viral GFP expression which only can be obtained in vital regions of the tissue samples (2.5 x/488 nm filter; white bars equate 1000 µM).

In order to approach our question in a patient-individual setting beyond isolated, cultured cells and cell lines, the *ex vivo* precision-cut tissue slice technology was adapted for our experimental situation [21]. Human liver tissue slices with a diameter of 0.8 cm and a thickness of 200–300 µm consisting of 10–15 cell layers, which harbor ∼10^6^ liver cells in average, were prepared from freshly resected liver tumors and corresponding non-malignant liver tissue, incubated in 24-well plates (Figure 4B). Samples of non-tumorous human liver tissues and human tumor tissues were of different origins (see tabular in Figure 4A) including hepatocellular carcinoma (HCC), colorectal carcinoma (CRC), pancreatic carcinoma (PC), and cholangiocarcinoma (CC). As viability control, precision-cut slices of human liver tissue (A; left column) and human tumor tissue (A; right column) were infected by default with a GFP marker gene encoding adenoviral vector (AdV-GFP, MOI 1) one hour after tissue cutting to determine the vitality of tissue slices cultured in 24-well plates. Only viable cells, exhibiting large areas being positive for GFP maker gene expression in both tumorous and non-tumorous primary human liver tissues provide the possibility for infection and subsequent expression of virus-encoded marker genes (note: tissue slices represent multi-cell layer pieces of surgically resected tissues (at a chosen slice thickness of 200–300 µm we calculate about 10–15 cell layers); therefore, it is technically not possible to bring all parts of the respective slices into focus (Figure 4B)).

As surrogate markers for cellular toxicity, (i) the amount of tissue-released LDH per mg protein, (ii) an ELISA-based determination of cytokeratin 18-cleavage correlating with caspase activity and, (iii) the caspase-induced DEVD cleavage activity per mg tissue protein were assessed.

**Figure 5:**
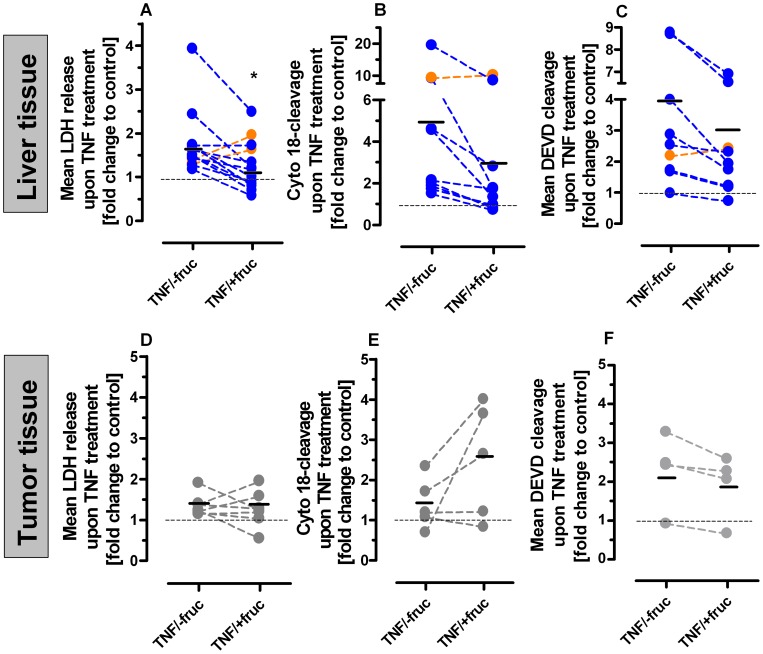
Abrogation of TNF-induced cell death under ATP depleting conditions in human liver tissues. Tissue slices derived from human livers (**A–C**) or liver tumor tissues (**D–F**), respectively, were treated with 1 µg/ml ActD in combination with 100 ng/ml TNF and 50 mM fructose as indicated. Cytotoxicity was determined by LDH release assay (**A/C**) and cytokeratin 18-cleavage (**B/D**) after 24 hours and by DEVD cleavage after eight hours (**C/F**). Data are depicted as mean fold changes to untreated control, being set to 1 (*: p<0.05, unpaired t-test).

Results obtained from tissue slices demonstrated the selective nature of fructose-mediated protection towards TNF also for patient-derived primary liver and liver tumor tissues. In primary non-malignant human liver tissue slices, the effect of TNF-induced hepatotoxicity on (i) LDH release (Figure 5A), (ii) cytokeratin 18-cleavage (Figure 5B), and (iii) DEVD cleavage (Figure 5C), respectively, was markedly diminished in presence of fructose in nearly all specimens. In contrast, in primary human liver tumor tissue slices, upon an identical pretreatment procedure with fructose, in most patient specimens no protection against TNF cytotoxicity was seen, if not even a sensitization towards TNF-induced cell death as demonstrated by levels of (i) LDH release (Figure 5D), (ii) cytokeratin 18-cleavage (Figure 5E), and (iii) DEVD cleavage (Figure 5F), respectively. The results derived from human tissue fits well into results obtained by utilizing isolated primary murine hepatocytes *in vitro* and a murine model of TNF-induced liver damage *in vivo* by Latta *et* *al*. [9]. TNF exerts a profound hepatic apoptosis and liver damage quantified by DEVD cleavage as well as by LDH and plasma ALT release, respectively. However, acute TNF-induced hepatotoxicity and liver damage in primary isolated murine hepatocytes and in mice, respectively, was entirely inhibited by ATP depletion with 50 mM fructose [9].

Consequently, we here show for the first time *ex vivo*, expelling cell culture artifacts and rodent epiphenomena, that adjunctive administration of fructose selectively protects primary human liver tissues *ex vivo*. In contrast, primary human tumor tissues derived from liver metastases are virtually not protected *ex vivo*. However, on individual levels, in some tumor patient-derived tissue specimens, fructose-loading failed to protect hepatocytes. It has to be kept in mind that endogenous pathologic conditions and disorders of the liver may alter fructose metabolism and sensitivity to TNF. Furthermore, tissue donors having received neo-adjuvant therapies could lead to alterations in the tumor's response prior to the putative onset of any TNF-based therapeutic regimen.

**Figure 6:**
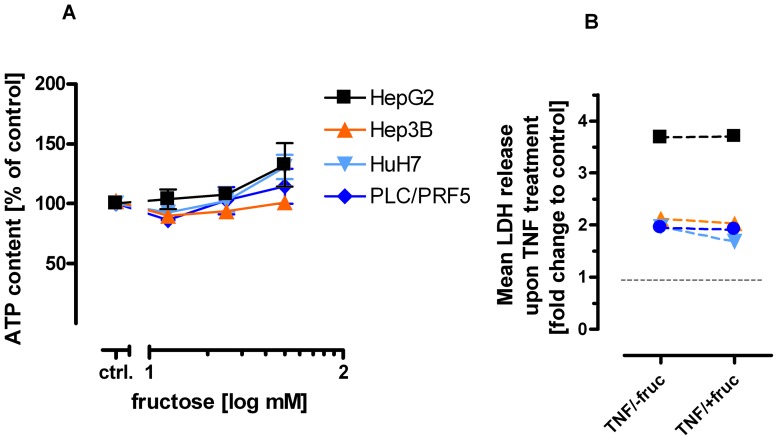
Neither ATP level nor cytotoxicity induction is depleted in hepatic tumor cell lines in presence of fructose. The hepatic tumor cell lines HepG2, Hep3B, HuH7 and PLC/PRF/5 were treated with increasing concentrations of fructose (**A**). ATP content was determined after 30 min (**A**) or at varying time points as indicated. Data are given as mean ± SEM (n = 15). The hepatic tumor cell lines were treated with 1 µg/ml ActD in combination with 100 ng/ml TNF and 50 mM fructose as indicated. Cytotoxicity was determined by LDH release assay after 24 hours (n = 15) (**B**). Data are depicted as mean fold changes to untreated control, being set to 1.

In analogy, in a set of human hepatic tumor cells – HepG2; Hep3B; HuH7 and PCL/PRF/5– neither an ATP depleting effect was induced by pretreatment with fructose (Figure 6A) nor any fructose-mediated protection towards TNF-induced apoptosis could be achieved (Figure 6B). Although the sensitivity towards TNF determined by means of LDH release assay was variable among the utilized tumor cell lines HepG2; HepB3; HuH7 and PLC/PRF 5, no mitigation of LDH release in presence of 50 mM fructose was yielded.

Comparing the transcriptional levels of glycolytic enzymes HKII, AldoB, and KHK, the mean level of HKII transcription was found to be increased in average 34-fold in the human hepatic tumor cell lines HepG2, Hep3B, HuH7 and PLC/PRF/5 in comparison to non-tumorous primary human liver tissues being normalized to factor 1. In contrast, enzymes AldoB and KHK were found to be constant or decreased in the examined tumor cell lines (mean fold changes <1 in comparison to non-tumorous primary human liver tissues being normalized to factor 1) as depicted in table 1. The enzymatic equipment of the tumor cells is highly indicative for the HKII bypass circumventing the ATP sink active in primary hepatocytes. Consequently, no ATP depletion was evident in presence of up to 50 mM fructose. Interestingly from a mechanistic point of view, in prior work it already has been demonstrated that the effects of fructose on ATP depletion and TNF-induced tumor cell death can be “artificially” reversed when primary murine hepatocytes, being “naturally” deficient for HKII like PHH, were gene complemented with a vector carrying the murine HKII gene [10]. In the study of Speicher *et* *al*. it was found that the physiological fructose/ATP trap was bypassed (like in liver tumor cells); as a result, the protection state “originally” achieved in primary hepatocytes under fructose loading now was lost, implementing a grossly enhanced sensitivity/toxicity of primary hepatocytes towards TNF-induced apoptosis [10].

## Conclusions

TNF constitutes a highly potent antineoplastic drug, which however so far cannot be applied to patients with primary and secondary tumors of the liver due to its profound hepatotoxicity. To overcome this obstacle, the combinatorial use of fructose *which can be used to exploit metabolic differences constituting a selective protection of healthy hepatocytes towards TNF* seems to be an interesting therapeutic option. Based on our results employing solely human primary hepatocytes and precision-cut slices of healthy human liver tissues *versus* malignant human liver tissues, it seems feasible that fructose-induced depletion of ATP represents a general way of protecting the human liver from unwanted energy-dependent cell death induced by TNF in IHP-based local cancer therapy whereby the sensitivity of liver tumors towards cytotoxic therapies is retained.
